# Prediction of Different Eye Diseases Based on Fundus Photography via Deep Transfer Learning

**DOI:** 10.3390/jcm10235481

**Published:** 2021-11-23

**Authors:** Chen Guo, Minzhong Yu, Jing Li

**Affiliations:** 1 Department of Computer and Data Sciences, Case Western Reserve University, Cleveland, OH 44106, USA; cxg451@case.edu; 2 Department of Ophthalmology, University Hospitals, Case Western Reserve University, Cleveland, OH 44101, USA

**Keywords:** eye diseases, multi-class classification, deep learning, transfer learning

## Abstract

With recent advancements in machine learning, especially in deep learning, the prediction of eye diseases based on fundus photography using deep convolutional neural networks (DCNNs) has attracted great attention. However, studies focusing on identifying the right disease among several candidates, which is a better approximation of clinical diagnosis in practice comparing with the case that aims to distinguish one particular eye disease from normal controls, are limited. The performance of existing algorithms for multi-class classification of fundus images is at most mediocre. Moreover, in many studies consisting of different eye diseases, labeled images are quite limited mainly due to privacy concern of patients. In this case, it is infeasible to train huge DCNNs, which usually have millions of parameters. To address these challenges, we propose to utilize a lightweight deep learning architecture called MobileNetV2 and transfer learning to distinguish four common eye diseases, including Glaucoma, Maculopathy, Pathological Myopia, and Retinitis Pigmentosa, from normal controls using a small training data. We also apply a visualization approach to highlight the loci that are most related to the disease labels to make the model more explainable. The highlighted area chosen by the algorithm itself may give some hints for further fundus image studies. Our experimental results show that our system achieves an average accuracy of 96.2%, sensitivity of 90.4%, and specificity of 97.6% on the test data via five independent runs, and outperforms two other deep learning-based algorithms both in terms of accuracy and efficiency.

## 1. Introduction

Fundus photography is a popular non-invasive method used to inspect anomalies associated with diseases of eyes and their progression, and has been widely utilized across the world for its comprehensiveness and convenience. Analysis of fundus images by ophthalmologists or optometrists is a labor-intensive task. With the ever-increasing quantity of fundus images as well as other types of images such as optical coherence tomography (OCT), there is a great shortage of human experts to interpret the available images, which results in delays of diagnoses and treatments [1]. In addition, readings based on fundus images from different doctors may not always be consistent. A final diagnosis of a patient often involves multiple doctors, which may further delay the process. Given the recent development in Artificial Intelligence (AI), especially in the area of deep learning and their successful applications in image analysis, machine learning systems that can automatically perform pre-clinical analysis and diagnosis can be a promising solution for the problem.

Recent development of machine learning methods for fundus image analysis is mostly focused on detection of one particular eye disease, and the problem is commonly formulated as a binary classification problem with the goal to separate disease images from normal images. The widely used approaches are mainly convolutional neural networks (CNN) with different network structures. For example, Dong et al. [2] built a five-layer CNN to identify cataract from fundus images and they showed that the results were better than some traditional machine learning methods such as Support Vector Machine. Burlina et al. [3] applied an eight-layer CNN called OverFeat to detect age-related macular degeneration (AMD). More recently, more complex networks such as deep CNNs have been created to classify AMD [4] and diabetic retinopathy [5]. In order to handle the challenge with small training datasets, the idea of transfer learning has been adopted, where pre-trained deep CNN models have been utilized in detecting glaucoma and have shown promising results [6,7].

Studying eye diseases one at a time based on fundus images is quite limited because fundus images can reveal many different conditions. However, the multi-disease classification problem based on fundus images is much harder. Research in this direction is very limited so far, and new methods are in great need given the inferior results from existing methods. For example, in an attempt to recover different sub-classes of AMD using ensemble learning based on six different convolutional neural networks, the study by Grassmann et al. [8] only obtained an overall accuracy of 63.3% on the AREDS dataset (http://dbgap.ncbi.nlm.nih.gov/, accessed on 19 October 2021). Multi-disease classification is even more challenging when the training dataset is small. Choi et al. [9] implemented an ensemble deep learning method to classify multi-categorical retinal images on a small dataset. They only achieved 31% accuracy when considering ten categories and 73% accuracy with three categories. Even with the help of transfer learning, the performance was only improved by 5% for the experiment with ten categories. The authors concluded that deep learning methods were not suitable for small datasets.

In this paper, we address the challenges of the multi-disease classification problem using small training data of fundus images, by taking advantage of some most recent breakthroughs in machine learning. The deep learning method of our primary focus is called MobileNetV2 [10], a lightweight deep neural network known for its high computing efficiency and excellent performance in image classification. To address the problem of a small training dataset, we also adopt the idea of transfer learning and first obtain a trained model based on ImageNet [11], followed by fine tuning of the model. Furthermore, we apply the gradient-weighted Class Activation Mapping (grad-CAM) [12] visualization method to highlight important areas the model utilizes to make predictions, a step towards the goal of explainable AI. We test the algorithm using a small dataset with 250 fundus images that consist of four different disease conditions (i.e., glaucoma, maculopathy, myopia, and retinitis pigmentosa) as well as normal controls. We compare its performance with two other deep learning approaches: InceptionV3 [13], which has been shown to be effective in multi-class learning of OCT images [14], and AlexNet [15], one of the first deep convolutional networks applied to fundus images [4]. Results show that MobileNetV2 achieves an average accuracy of 96.2% on the test data over five independent runs, outperforming both InceptionV3 and AlexNet. Visualization results also show that the model mostly focuses on the areas that are relevant to each specific disease.

The rest of the paper is organized as follows. We introduce the model structure in Section 2, as well as details of transfer learning, visualization, and alternative approaches. The description of the dataset, the prediction results, the comparison with the alternative approaches, and visualization results can be found in Section 3. We conclude the paper with brief discussions and possible future work in Section 4.

## 2. Materials and Methods

In this section, we first introduce the network structure of MobileNetV2 and the training details. In combination with transfer learning, the system explicitly addresses the challenges of the multi-class classification problem with small training data. We also explain the visualization algorithm and discuss the two other approaches, InceptionV3 and AlexNet, in this section.

### 2.1. MobileNetV2 Model Architecture

**Figure 1:**
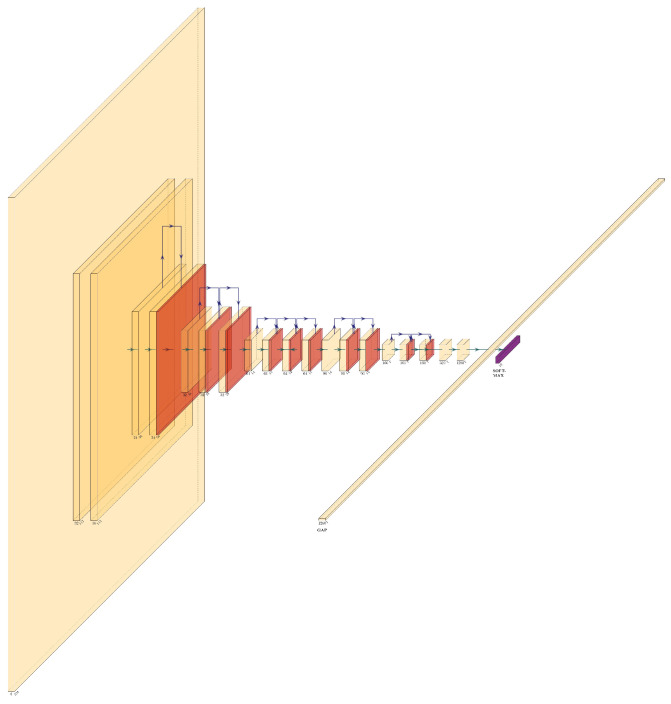
The network architecture of MobileNetV2, including the input layer, 19 bottleneck layers, the average pooling layer, and the prediction layer. A link on top of two adjacent blocks indicating the residual shotcut and the corresponding output block is colored differently using dark brown. Due to the size of the figure, the feature dimension is not scaled.

Figure 1 shows the structure of our model using MobileNetV2 as the feature extractor. The size of the input layer is 224×224×3, followed by 1 fully convolution layer with 32 filters. The main structure of the network consists of 17 residual bottleneck layers and blocks (Sandler et al. [10]), followed by 1 global average layer and the prediction layer which utilizes the softmax activation function. Each reverted residual block consists of one 1×1 convolutional kernel that expands the feature maps, followed by a 3×3 depthwise convolutional layer that generates the same number of feature maps as the inputs, and another 1×1 convolutional layer to recover the output shape to a lower dimension again. A shotcut (residual) is applied between two adjacent blocks if they have the same dimension and the stride is 1. The ReLU6 activation and the batch normalization are applied inside each block (Sandler et al. [10]).

The design of MobileNetV2 focuses on the reduction in the size of the network and at the same time, it tries to maintain prediction accuracy. It has a lower parameter/layer ratio. The number of parameters in the deep separable convolution of each block can reduce to around 30% of the normal convolution operations. The residual connection in MobileNetV2 can also solve the gradient vanishing problem, which in turn improves prediction accuracy. Furthermore, the inverted residual of MobileNetV2 allows the dimension in each block to follow the narrow-wide-narrow (i.e., bottleneck) pattern, which achieves better memory utilization according to the original paper (Sandler et al. [10]).

### 2.2. Model Training and Transfer Learning

We implemented the MobileNetV2 [10] feature extractor using the Tensorflow environment and the training was performed on the Google Colab platform with GPU runtime. The objective function is the categorical cross-entropy loss, and we used the RMSprop library for optimization. The instantiates of the feature extractor and its pre-trained weights based on ImageNet dataset were obtained from Keras Applications [16]. The training consists of two steps: the transfer learning step and the fine tuning step. During the phase of transfer learning, most of the pre-trained weights obtained from ImageNet are frozen, which not only avoids overfitting due to small training data, but also allows fast convergence. The only weights that are learnable are those between the last convolutional layer and the final prediction layer. The number of learnable parameters is around six thousand out of more than two million parameters. For the fine tuning step, we further unfroze the parameters between the last two convolutional layers, with the total number of trainable parameters close to nine thousand.

### 2.3. Visualization of Important Areas in Distinguishing Different Classes

To better understand and locate areas that contribute the most to the classification results, we utilize the gradient-weighted Class Activation Mapping (grad-CAM) [12] visualization method to show important areas and to examine whether the identified areas make biological sense in defining different diseases. The basic idea of grad-CAM is to use the gradients of any target concept flowing into the final convolutional layer to produce a coarse localization map highlighting the important regions in the image for predicting the concept, and it can be applied to many CNN architectures [12].

By unfreezing the parameters in the last several layers during the transfer learning and fine tuning, the weights learned are calculated based on our own image data. This allows the establishment of connections between the feature map of the last convolutional layer and the fundus images, which enables grad-CAM to actually visualize the important areas of the input fundus images. To actually visualize the areas, a new weight for each feature map is calculated based on the gradients to the predicted class and a heat map is computed by summing the weights from all feature maps of the last convolutional layer. Through upsampling, the generated heat map is the same size as the original image, and the location that contributes to the predicted label can be highlighted.

### 2.4. Alternative Approaches

As a comparison, we also include two additional deep learning models in our study: AlexNet and InceptionV3. AlexNet was one of the earliest DCNNs that was very successful for image classification in the ImageNet contest [15]. It consists of eight layers with five convolutional layers and three fully connected layers. The number of parameters is more than 60 million. We chose AlexNet as one of the alternative approaches because it was successfully applied in detecting age-related macular degeneration from fundus images [4]. InceptionV3 is a 42-layer DCNN that utilizes several techniques to reduce the total number of parameters and to speedup the calculation [13]. It performed very well for image classification in the ImageNet Large Scale Visual Recognition Competition in 2015 [13]. More recently, researchers have utilized the pre-trained InceptionV3 network for classification of multiple eye diseases based on retinal OCT images and have achieved performance comparable to that of human experts [14].

For both AlexNet and InceptionV3, we followed the network configuration and training details outlined in the two papers [4,14]. More specifically, AlexNet is optimized via the Stochastic Gradient Descent algorithm with Nesterov Momentum. The initial learning rate is 0.001 and the total number of epochs is 1000. The training of InceptionV3 is based on the Adam optimizer. The number of epochs is 16 with an initial learning rate of 0.001. Because transfer learning is also used in InceptionV3 [14], we also performed fine-tuning (unfreeze the weights between the last two convolutional layers for additional 30 epochs) to improve its performance.

## 3. Results

### 3.1. Data Statistics

The medical images were obtained from a public dataset on Kaggle (https://www.kaggle.com/linchundan/fundusimage1000/, accessed on 19 October 2021). We used a subset of the dataset in this study, consisting of four diseases and the normal eye images, which was verified by retinal specialists. The entire dataset contains only 250 fundus images from five classes (75 glaucoma images, 72 images labeled with maculopathy, 49 myopia, 22 retinitis pigmentosa, and 32 images showing an absence of any diseases, labeled as normal). We separated the data into training (80%) and testing (20%) for each category, thus the training set contains 202 samples and the remaining 48 samples are for testing. The resolution of the original images is very high (around 2000 × 3000). They are reshaped to the same size of 224 × 224 to fit the pre-trained networks. The color information from the original data is maintained.

### 3.2. Model Training and Hyper-Parameters

**Figure 2:**
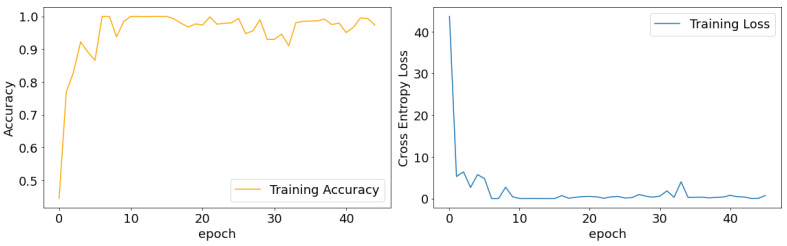
The training accuracy and training loss curves during the transfer learning step.

As we mentioned earlier, the training of our model consists of two steps: the transfer learning step and the fine tuning step. In the transfer learning step, the only parameters learnable are the weights between the last convolutional layer and the final prediction layer. The model converges quickly within several epochs (Figure 2), which shows the advantage of transfer learning in speeding up the training process. After 16 iterations, the training loss and accuracy do not change much. Therefore, for the rest of the experiments, we set the number of epochs to 16, with 4 samples from each batch and an initial learning rate of 0.1. For the fine tuning step, we further unfroze two more layers of the feature extractor and set the epoch to 30 with a initial learning rate of 0.01.

### 3.3. Prediction Results

**Figure 3:**
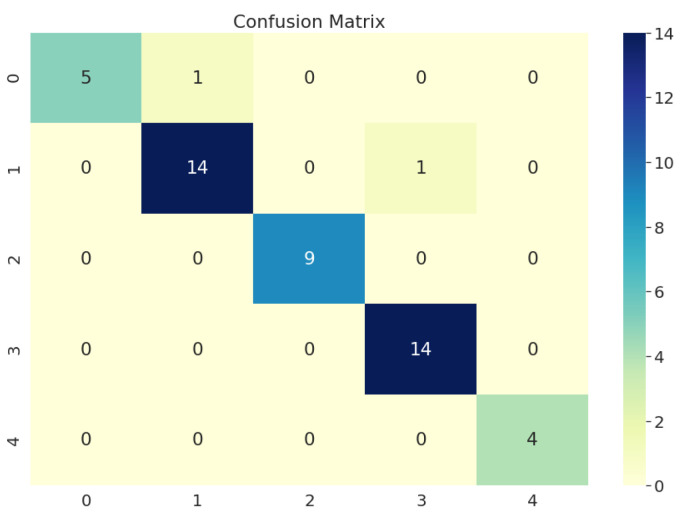
The confusion matrix of the multi-class classification results by MobileNetV2 on the test dataset. The class labels: 0 for healthy control, 1 for glaucoma, 2 for pathological myopia, 3 for maculopathy, and 4 for retinitis pigmentosa.

After obtaining the trained model, we applied the model to the remaining 20% test data. The model was successful in distinguishing all five classes: glaucoma, maculopathy, pathological myopia, retinitis pigmentosa, and healthy control. It achieved a remarkable 98% in accuracy, 95.8% in sensitivity, and 99.0% in specificity. The confusion matrix is shown in Figure 3. The results illustrate that with transfer learning, MobileNetV2 can achieve incredible results in classifying different eye diseases based on fundus images with a very small amount of labelled data.

**Figure 4:**
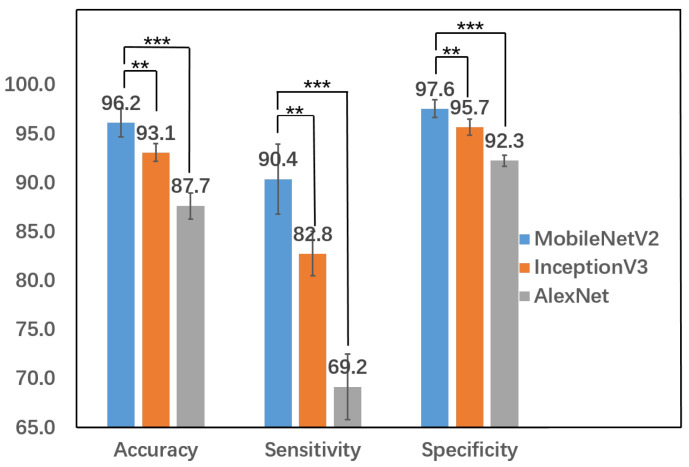
The performance comparison using the *t*-test for the three approaches on five independent runs. **: significant at *p*-value 0.01. ***: significant at *p*-value 0.0001.

To compare the performance of the three different models, we performed the following experiments in five independent runs. In each run, the whole dataset was randomly split into training (80%) and testing (20%). Each model was trained on the training dataset and results of the test dataset are shown in Table 1. Clearly, MobileNetV2 outperformed InceptionV3 and AlexNet in all the measures. It achieved an average accuracy of 96.2% (±1.4%), sensitivity 90.4% (±3.6%), specificity 97.6% (±0.9%). To assess the statistical significance of the performance differences in comparing MobileNetV2 vs. InceptionV3 and AlexNet, we performed a double-tailed *t*-test on all three measures. Furthermore, Levene’s test was performed to compare the variance before applying the *t*-test. For all the three measures and for both comparisons (MobileNetV2 vs. InceptionV3, and MobileNetV2 vs. AlexNet), the *p*-values of Levene’s test are in the range of 0.79 to 0.81. Therefore we cannot reject the null hypotheses that have equal variance, and the *t*-test can be used to compare the means. MobileNetV2 performs significantly better than InceptionV3 and AlexNet (Figure 4), with a *p*-value of $3.9\times 10^{-3}$ for all three measures for the former and a *p*-value $1.08\times 10^{-5}$ for all the three measures for the latter. In addition, it is twice as fast as InceptionV3 and 40 times faster than AlexNet. Other than the differences in network structures, both MobileNetV2 and InceptionV3 utilize transfer learning and both of them perform much better than AlexNet in terms of accuracy and efficiency, indicating the applicability and importance of transfer learning in classifying small medical image data using deep learning approaches.

### 3.4. Towards Explainable AI-Based Diagnosis

**Figure 5:**
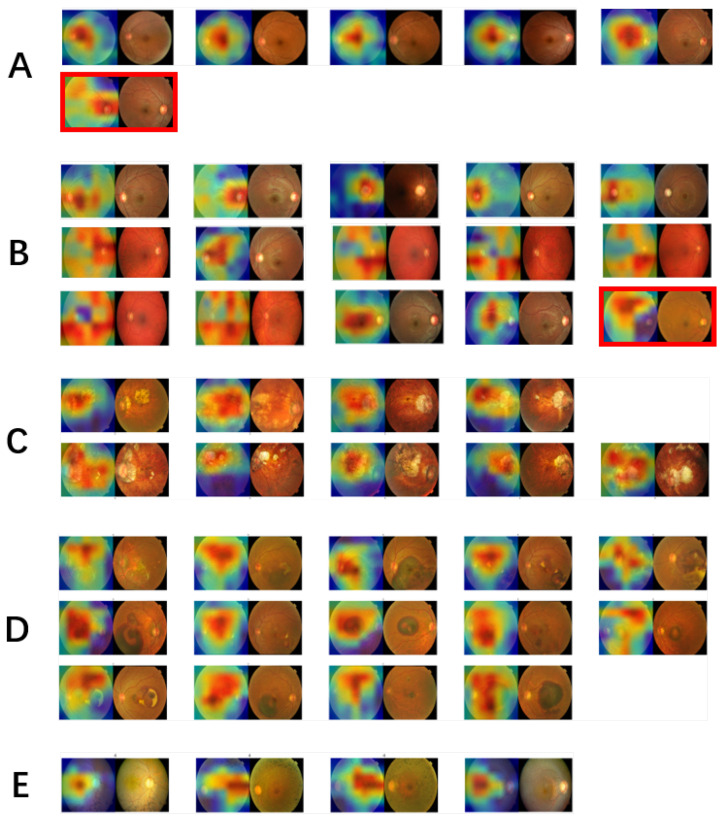
The original images in the test dataset and their corresponding heatmaps generated by our algorithm showing the weight distribution contributing to the classification of different labels. Each of the two misclassified images is marked with a red frame around it. The true class labels are: (**A**) for Normal, (**B**) for Glaucoma, (**C**) for Pathological Myopia, (**D**) for Maculopathy, and E for Retinitis Pigmentosa.

In clinical diagnosis of eye diseases based on fundus images, different regions and different characteristics/features are examined and used by ophthalmologists or optometrists. For example, it is commonly known that the cup-to-disc ratio at the optic disc is used to identify glaucoma. On the other hand, Fuchs spot, tigroid fundus, lacquer cracks (yellow, yellow-white spots or bands), and a tilted disc can be found in fundus images with pathological myopia. As we mentioned earlier, by taking advantage of the grad-CAM method, we can create a heatmap and visualize the important areas on each image that our method utilizes to classify different diseases. Doing so is a first step towards the ultimate goal to build an explainable AI-based diagnosis system. Therefore, for all the test images, we show the original images and their heatmaps side by side in Figure 5 to examine the biological relevance of the regions that received higher weights in MobileNetV2.

Overall, for the majority of the images for all four diseases (Figure 5B–E), the highlighted areas (warm color) are important in distinguishing different types of diseases. For example, Figure 5B shows the images with glaucoma. For seven out of the fifteen images, their optic discs are covered by highlighted areas (the first seven small images in Figure 5B). For the next five images, the highlighted areas are located around the optic disc. On the other hand, for the misclassified one (the last with a red frame, misclassified as maculopathy), the highlighted areas are far from the optic disc. Figure 5C shows for all pathological myopia images, our model was able to locate Fuchs spot areas. A maculopathy is any pathological condition related to the macula, an area at the centre of the retina. And Figure 5D shows that for all the images with maculopathy, our algorithm largely considered the central area of the retina. Retinitis pigmentosa is usually diagnosed with dark pigments in the peripheral regions of the retina. Figure 5E shows that the dark pigments were highlighted in the peripheral area of the images with retinitis pigmentosa, and the pigmentary changes or drusen were captured in all the four images. In terms of the normal images (Figure 5A), the algorithm focused mainly on the central areas for all but one, for which the algorithm focused on the disc area, and the image was misclassified as glaucoma.

### 3.5. Binary Classification

**Figure 6:**
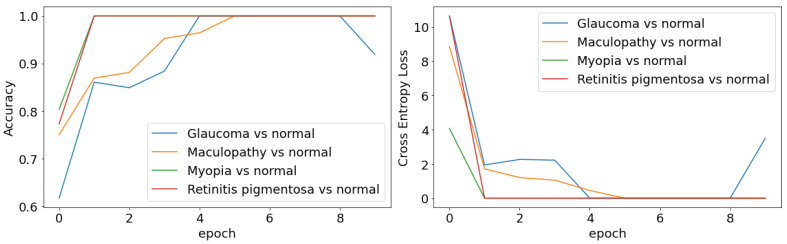
Learning curves of the binary classifiers.

We also tested our algorithm as a binary classifier on the same dataset, which goal was to differentiate each individual disease from normal controls. For each of the diseases, we coupled the images with the control samples and separated data into training (80%) and testing (20%). As expected, the problem became easier and the algorithm learned very quickly in a few epochs (Figure 6). For both pathological myopia and retinitis pigmentosa, the algorithm achieved perfect predictions for both the training and the testing data (Figure 6 and Table 2). Additionally, for both glaucoma and maculopathy there was only one misclassified instance.

## 4. Discussion

In this paper, we study the problem of retinal disease diagnosis based on fundus images using AI and machine learning algorithms. Our goal is to correctly label each image with one of several diseases. One of the challenges in the analysis is the limited number of training images. We propose to utilize the transfer learning technique and take advantage of the previously trained deep learning model MobileNetV2. Our results on a small dataset with 250 fundus images and five different labels show that MobileNetV2 was able to correctly predict most of the images and achieved an average accuracy of 96.2%. It outperformed two other alternative deep learning models, AlexNet and InceptionV3, both in terms of accuracy and efficiency. Our study shows the capacity of deep learning in the field of fundus image diagnosis, and transfer learning makes training on a small dataset a reality. The fine-tuning step further improves the performance of the model.

In an attempt to overcome the drawbacks of black box AI models, we add the explainable learning step by highlighting the areas on the images that are important to the classification results, based on the learning process. The overall results based on the visualization show some promising progress and the areas of focus are mostly aligned with the disease labels. At the same time, we observe that for some images, the highlighted areas are different from where they are supposed to be based on the opinions of human experts. This kind of gap seems inevitable given the small dataset. As a future direction, we will investigate possible ways to “guide” the algorithms to focus on important areas or features. One possible direction is to incorporate prior knowledge into deep learning models, a topic that has recently drawn attention from the deep learning community [17]. Although deep learning enjoys great success with end-to-end learning with no prior knowledge, for small datasets, similar to transfer learning, incorporating prior knowledge can potentially play an important role in achieving better results, especially for medical image data where forward predictions require reasoning.

When MobileNetV2, as well as many other deep learning models, were trained on the ImageNet dataset, they normally resized their input images to the size of 224 × 224. The network structures and parameters were optimized based on the input size. In this study, the original images have very high resolutions (e.g., of size ∼2000 × 3000). To fit the data into the pre-trained models, we simply downsampled the images. Doing so obviously loses some information retained in the high resolution images. In theory, the models can handle images of different sizes if the models are trained from scratch and if sufficient computational resources are available. For transfer learning, although one can possibly change the dimension of the input tensor to accommodate images of different sizes in practice, the use of such an strategy has certain limitations. In models with fully connected layers, the weights may not be transferable. In addition, when the images are too small, they may not provide enough data through many pooling layers. When the images are too large, the network architectures may not have enough number of layers to learn reliable and discriminative filters. In either case, fine-tuning, or even modification of the network structures are needed. This essentially is a trade off between computational costs and prediction accuracy. Our experiments show that downsampling the images beforehand produced quite robust results with high accuracy. Nevertheless, we will explore the impact of different options on prediction accuracy in the future.

Fundus photography allows clinicians to diagnosis many eye diseases and conditions, and to monitor their progression. In theory, the proposed model can be extended easily to incorporate many more different conditions/labels. Further experiments are needed to assess how well the model will perform with more images of different disease classes added. In this study, each image is labeled with one disease. In practice, one patient might have more than one eye disease. The computational problem is called multi-label learning, and it is generally harder than the multi-class learning proposed here. Furthermore, images with multiple labels (i.e., training data) are generally hard to obtain. Nevertheless, this topic can be considered for future studies.

This study only consists of monoscopic fundus images. Fundus cameras have the ability to stereoscopically image the central and peripheral retina by sequentially taking two images. It would be interesting to extend the system to incorporate stereoscopical images and compare the performance using these two different types of images. This will be one of our future research directions. Furthermore, clinicians rarely make a diagnosis purely based on fundus images. Instead, multiple evidences are usually considered when making a decision (e.g., eye pressure is used together with fundus images for the diagnosis of glaucoma). In the future, we can take advantage of multi-module learning by constructing models that can take different types of features as input to better capture the information clinicians use in real clinical settings.

## Figures and Tables

**Table 1:** Experimental results of five independent runs from the three methods.

**Accuracy (%)**							
Method	1	2	3	4	5	Mean	StdDev
AlexNet	88.0	86.4	89.6	88.0	86.4	87.7	1.3
InceptionV3	93.6	92.0	92.8	94.4	92.8	93.1	0.9
MobileNetV2	96.0	94.4	98.4	96.0	96.0	96.2	1.4
**Sensitivity (%)**							
Method	1	2	3	4	5	Mean	StdDev
AlexNet	70.0	66.0	74.0	70.0	66.0	69.2	3.3
InceptionV3	84.0	80.0	82.0	86.0	82.0	82.8	2.2
MobileNetV2	90.0	86.0	96.0	90.0	90.0	90.4	3.6
**Specificity (%)**							
Method	1	2	3	4	5	Mean	StdDev
AlexNet	92.5	91.5	93.5	92.5	91.5	92.3	0.5
InceptionV3	96.0	95.0	95.5	96.5	95.5	95.7	0.8
MobileNetV2	97.5	96.5	99.0	97.5	97.5	97.6	0.9
**Time (s)**							
Method	1	2	3	4	5	Mean	StdDev
AlexNet	977.11	992.42	1001.24	945.09	918.87	966.95	34.340
InceptionV3	50.99	53.19	51.90	52.49	53.37	52.39	0.974
MobileNetV2	23.35	24.90	24.21	25.01	25.04	24.50	0.728

**Table 2:** Prediction results of the binary classifiers on test data.

Metric	Glaucoma	Maculopathy	Pathological Myopia	Retinitis Pigmentosa
Accuracy	95.2	95.0	100.0	100.0
Sensitivity	100.0	100.0	100.0	100.0
Specificity	83.3	83.3	100.0	100.0

## Data Availability

Data Availability Statements: the code and datasets are available at https://github.com/gcowen/fundusimageclassification, accessed on 19 October 2021.

## Abbreviations

The following abbreviations are used in this manuscript:

DCNN	Deep convolutional neural network
OCT	Optical coherence tomography
AI	Artificial Intelligence
CNN	Convolutional neural networks
AMD	Age-related macular degeneration
grad-CAM	Gradient-weighted class activation mapping
